# Mechanisms influencing group teaching learning achievement in university students: the mediating role of social loafing

**DOI:** 10.3389/fpsyg.2026.1745663

**Published:** 2026-07-13

**Authors:** Yuyan Cong

**Affiliations:** School of Education, Taiyuan Normal University, Taiyuan, China

**Keywords:** group teaching, learning achievement, learning motivation, peer support, rating fairness, self-efficacy, self-expectation, social loafing

## Abstract

In modern higher education, group teaching is widely used to enhance students' academic achievements and comprehensive abilities. However, social loafing in group activities remains a pressing issue. Grounded in Achievement Goal Theory, this study aims to explore how internal mastery-oriented factors and contextual goal structures jointly influence social loafing and subsequent learning achievement. Specifically, it examines the impact of self-efficacy, learning motivation, self-expectation, peer support, and rating fairness on social loafing and their subsequent effect on learning achievement. Through a survey and data analysis of 600 university students, the results indicate that self-efficacy and learning motivation significantly positively influence self-expectation; peer support and rating fairness significantly reduce social loafing, whereas self-expectation does not show a direct inhibitory effect; and social loafing negatively affects learning achievement. Additionally, rating fairness and peer support significantly enhance learning achievement by reducing social loafing. This study not only validates and extends Achievement Goal Theory in collaborative learning contexts but also indicates that in group teaching, internal motivation must be transformed through self-expectation and contextual goal structures to effectively inhibit social loafing, integrating individual and instructional factors while highlighting the critical roles of rating fairness and peer support.

## 1 Introduction

Group teaching is an important instructional strategy in higher education, as it has been shown to increase students' academic success, enhance communication, teamwork, and critical thinking (Rajaram and Rajaram, 2021). To be successful, group teaching must include clearly defined roles, appropriate teacher support and direction, and effective methods of managing group dynamics (Herrera-Pavo, 2021). Students' motivation is diminished when they feel that they have not received an equitable evaluation or are not recognized (Arianto and Yasin, 2023), while peer evaluation can increase motivation and engagement (Vander Schee and Birrittella, 2021).

One major issue associated with group learning is social loafing. Social loafing occurs when an individual's contribution to a group effort diminishes due to low motivation, thus impacting both work performance and learning performance (Lam, 2015; Piezon and Donaldson, 2005). Previous research has identified several factors contributing to social loafing within teams, such as team size, task requirements, and evaluation methods (Aggarwal and O'Brien, 2008; Rajaguru et al., 2020). However, previous studies tend to emphasize structural conditions heavily and have not adequately considered students' internal motivational perspectives within higher education settings. Similarly, research on group achievement in educational settings tends to focus on instructional or evaluative design rather than on the processes through which motivation influences achievement. Furthermore, there is a deficit in the literature that relates social loafing as a mediating process through which motivation can influence group achievement.

Additionally, research on motivation and social loafing typically occurs in isolation from one another. Motivation research focuses primarily on self-efficacy and motivation, whereas social loafing research is largely framed as a social-psychological or organizational phenomenon. Therefore, there is currently no theoretical integration of motivation and social loafing that demonstrates how internal motivation and external perceptions of group learning can interact to create a propensity for social loafing and influence group achievement.

In light of these findings, this study will use Achievement Goal Theory (AGT) as its theoretical framework. Within this framework, self-efficacy, self-expectation, and motivation are viewed as internal factors that guide students in setting and pursuing goals. At the same time, peer support and the perceived fairness of ratings will be considered key contextual influences on goal setting and achievement. The AGT framework will also provide a conceptual perspective on social loafing as a product of reduced engagement with mastery-oriented goals and external influences, depending on motivational and contextual factors.

The purpose of the proposed research study is to identify the psychological antecedents of social loafing, investigate the mediating effect of social loafing on group achievement, and to integrate isolated streams of motivation and group learning research. By providing a clearer understanding of the antecedents of social loafing, this study will make a theoretical and practical contribution to improving the effectiveness of group teaching in higher education.

## 2 Literature review

### 2.1 Achievement goal theory

AGT originates from the social cognitive approach to the study of learning motivation and emphasizes the central role of achievement goals in individual motivation. Ames (1992) defined achievement goals as the purposes of achievement behavior, referring to an integrated pattern of beliefs, attributions, and affects that shapes behavioral intentions. This theory posits that learning outcomes are influenced not only by ability but also by goal orientation, self-regulation, and persistence (Dever et al., 2024; Gao and Brown, 2023). As a motivational theory, AGT encompasses cognitive, affective, and behavioral characteristics (Bardach et al., 2020; Urdan and Kaplan, 2020) and integrates personal and contextual motivational factors (Cheng, 2023), explaining how learners construct subjective meaning within learning environments (Kaplan and Patrick, 2016). In the context of group teaching, learners' perceptions of role allocation, responsibility attribution, and evaluation fairness also constitute important contextual factors that influence goal adoption and the quality of motivation.

AGT distinguishes between mastery goals and performance goals (Elliot and Hulleman, 2017). Mastery goals emphasize competence development and deeper understanding and are associated with deeper cognitive engagement and persistent strategic behavior (Elliot and Hulleman, 2017). Performance goals focus on social comparison and the demonstration of competence, and excessive emphasis on performance may reduce motivation among some learners (Ding and Wang, 2025). Different goal types elicit different affective and behavioral responses, thereby influencing learning outcomes (Zhao, 2025). In group teaching, when instructional design emphasizes the collaborative process, clearly defined role allocation, and active guidance, and enhances perceptions of fairness through peer evaluation, mastery orientation can be strengthened (Vander Schee and Birrittella, 2021). In contrast, when task allocation is imbalanced or effort is not evaluated fairly, motivation and participation may be weakened (Arianto and Yasin, 2023).

This study adopts AGT as its theoretical foundation. Self-efficacy, learning motivation, and self-expectation are categorized as personal motivational factors that represent core belief components supporting mastery goal orientations. These constructs were selected because AGT emphasizes competence beliefs and goal meaning construction as central mechanisms underlying goal adoption and sustained effort. Rather than including all theoretically related constructs (e.g., self-regulated learning, competitive climate), this study focuses on belief variables that are more directly linked to effort investment and behavioral engagement in collaborative tasks.

Peer support and equitable grading constitute important contextual factors within the group teaching context and function as classroom goal structure cues rather than being treated simply as performance goals. According to AGT, classroom structures communicate implicit standards of competence and responsibility. These contextual signals were selected because social loafing in group teaching is closely associated with responsibility diffusion and effort visibility; therefore, peer interaction norms and evaluation transparency are more directly relevant to the behavioral manifestation of disengagement than other contextual variables such as general classroom climate or teacher support.

When learners internalize mastery-oriented engagement supported by competence beliefs and meaningful goals, they are more likely to actively participate in group tasks and reduce social loafing. Conversely, when classroom goal structures fail to reinforce responsibility or fairness, the tendency toward social loafing may increase. Therefore, the integration of representative personal belief components and contextual goal structure cues within AGT provides a coherent theoretical basis for the variable framework of this study and the mechanism of social loafing.

### 2.2 Self-expectation

Self-expectation refers to an individual's belief and expectation regarding their performance capability in specific tasks or behaviors. These beliefs not only reflect the individual's assessment of task difficulty and their abilities but also include expectations for future performance (Meyer et al., 2019). The primary goal of self-expectation is to enhance an individual's confidence and motivation when facing academic tasks, thereby improving academic achievement. It indirectly promotes academic performance by influencing the individual's learning motivation and persistence (Doménech-Betoret et al., 2017). Research indicates that self-expectation significantly impacts academic performance. Students with high self-expectation perform better in academic tasks and are more likely to persist in completing their studies (Zhang et al., 2024). Additionally, self-expectation can stimulate students' intrinsic learning motivation, making them more proactive when facing learning tasks (Deci et al., 1991). Furthermore, self-expectation helps students remain persistent in the face of difficulties, preventing them from giving up easily and enabling them to achieve long-term learning goals (Zimmerman, 1995). In summary, self-expectation plays a critical role not only in enhancing academic performance but also in stimulating learning motivation and promoting continuous learning.

### 2.3 Self-efficacy

Self-efficacy refers to an individual's belief in their ability to organize and execute actions in specific situations. This belief affects the choice of tasks, the effort put into them, the persistence in facing difficulties, and the reactions to challenges. According to Bandura's social cognitive theory, self-efficacy originates from personal accomplishments, vicarious experiences, verbal persuasion, and physiological states (Bandura, 2001). The purpose of self-efficacy is to enhance an individual's confidence in their abilities, promoting success in academic and life endeavors. High self-efficacy can increase students' learning motivation, helping them remain persistent when facing challenges, thereby achieving higher academic success (Schunk, 1984). Research shows that self-efficacy significantly predicts students' academic performance. Students with strong self-efficacy perform better academically and are more likely to complete their studies (Lane and Lane, 2001). Additionally, students with high self-efficacy demonstrate greater motivation and engagement in the learning process, which helps them participate more actively in learning activities (Zimmerman, 2000). Furthermore, self-efficacy can indirectly enhance learning outcomes by influencing students' perceptions of the learning environment. Supportive teaching and a positive classroom atmosphere significantly contribute to enhancing students' self-efficacy (Lorsbach and Jinks, 1999). Finally, students with high self-efficacy are better at self-regulated learning, managing their time, setting goals, and monitoring their progress, which helps improve learning outcomes (Meyer et al., 2019).

Improving self-efficacy can significantly enhance students' confidence in their academic abilities, thereby strengthening their self-expectation. Empirical evidence indicates a significant correlation between self-efficacy and students' academic achievement and persistence, with students demonstrating higher levels of self-efficacy performing better in academic tasks (Zhang et al., 2024). In addition, students with high self-efficacy exhibit stronger coping abilities when facing academic pressure, maintaining elevated levels of self-expectation and academic goals (Wood et al., 2015). From the perspective of AGT, individuals' self-efficacy influences their achievement goal orientation, which in turn shapes their learning behaviors and affective responses (Ames, 1992; Elliot and Hulleman, 2017). Another study indicates that college students' self-efficacy significantly affects their academic integration and adaptability, with students possessing higher self-efficacy demonstrating stronger adaptability and academic achievement in the university environment (Wood et al., 2015). According to AGT, when students possess higher self-efficacy, they are more likely to adopt a mastery goal orientation that emphasizes competence development and sustained engagement, thereby enhancing their self-expectation and academic development (Zhao, 2025).

Recent studies also support this view. For example, Muchtar et al. (2023) found that students with high self-efficacy not only perform better academically but also maintain better mental health when facing academic pressure. Additionally, Shofiah et al. (2023) showed that academic self-efficacy mediates the relationship between academic motivation and academic achievement, further emphasizing the crucial role of self-efficacy in promoting academic success. In summary, self-efficacy significantly enhances students' self-expectation and academic achievement by boosting their confidence and coping abilities, promoting academic success. This study proposes the following hypothesis:

H1: University students' self-efficacy positively influences their self-expectation.

### 2.4 Learning motivation

Learning motivation refers to the internal psychological state and driving force that initiates, directs, and sustains individuals' engagement in learning activities. It functions to stimulate and maintain learners' behavior toward specific academic goals (Filgona et al., 2020). Consistent with this view, learning motivation has been conceptualized as an internal process that guides learning purposes, regulates learning behaviors, and reinforces students' psychological engagement in academic tasks (Lei et al., 2024). It reflects students' inner drive and psychological state that influence academic performance (Lei et al., 2024), emerging from internal needs and desires that prompt individuals to initiate or persist in goal-directed behavior (Pelikan et al., 2021). As an internal driving force, variations in learning motivation are associated with differences in students' level of engagement and investment in learning (Sánchez-Bolívar and Martínez-Martínez, 2022). High levels of learning motivation help students set higher learning goals and maintain persistence when facing difficulties, thereby achieving higher academic success (Yang and Wang, 2022). Learning motivation is significantly associated with academic achievement, with highly motivated students demonstrating better task performance and a greater likelihood of attaining their learning goals (Wardani et al., 2020). Moreover, motivation serves as a key driver of lifelong learning, as students with stronger learning motivation are more likely to sustain continuous self-improvement in knowledge and skills (Gopalan et al., 2017). Although prior literature often distinguishes intrinsic and extrinsic dimensions of motivation, the present study conceptualizes learning motivation as a general, composite construct reflecting students' overall internal drive to engage in learning rather than differentiating specific motivational types.

Learning motivation is a critical factor influencing university students' self-expectation. Enhancing learning motivation strengthens students' confidence in their academic abilities, thereby improving their self-expectation. According to AGT, learning motivation reflects students' construction of the subjective meaning of achievement activities, and goals define the purpose and value of their engagement in learning (Kaplan and Patrick, 2016; Cheng, 2023). When students perceive learning as a process of growth and competence development, their self-expectation correspondingly increases. Empirical studies have identified significant relationships among learning motivation, academic achievement, and self-efficacy. Students with higher levels of learning motivation perform better in academic tasks and are more likely to attain their academic goals (Yang and Wang, 2022). In addition, learning motivation indirectly enhances self-expectation by strengthening students' self-regulation abilities. Highly motivated students are more capable of employing effective learning strategies, managing time, setting goals, and monitoring progress, thereby improving academic achievement and self-expectation (Yang and Wang, 2022). Furthermore, AGT indicates that different achievement goals elicit distinct affective and behavioral responses, which subsequently influence learning outcomes and future expectations (Elliot and Sommet, 2023; Zhao, 2025). Therefore, higher levels of learning motivation contribute to the development of positive goal orientations and reinforce self-expectation. Taken together, learning motivation significantly enhances university students' self-expectation and academic achievement by strengthening confidence and self-regulation abilities, thereby promoting academic success. This study proposes the following hypothesis:

H2: University students' learning motivation positively influences their self-expectation.

### 2.5 Social loafing

Social loafing refers to the phenomenon where individuals exhibit lower levels of effort when working in a group task compared to working alone. This phenomenon often occurs in group settings when an individual's personal contributions are difficult to identify, leading them to reduce their effort (Yasegnal and Tolla, 2025). The primary aim of researching social loafing is to understand its causes and effects to implement effective measures to reduce its negative impact in work and educational environments. Analyzing individual performance in group tasks can help organizations and educational institutions design more effective team collaboration mechanisms, thereby enhancing overall efficiency. Understanding social loafing can help identify and address inefficient behaviors in team work, thereby improving overall team efficiency. Measures to reduce social loafing include increasing task visibility and individual accountability (Liden et al., 2004). Additionally, in businesses and organizations, social loafing can lead to poor employee performance, affecting overall productivity. By understanding and reducing social loafing, organizations can improve employee performance and enhance competitiveness (Turksoy, 2020). Finally, effectively managing and reducing social loafing can promote cooperation and trust among team members, thereby strengthening team cohesion.

Self-expectation can effectively inhibit social loafing among university students in group learning contexts. When students possess high self-expectation, they are more likely to establish elevated standards for their academic performance and actively strive to achieve these standards, thereby reducing the tendency to decrease individual effort in the presence of others during group tasks, a phenomenon defined as social loafing. From the perspective of AGT, achievement goals shape individuals' behavioral choices and perceptions of responsibility within group settings (Ames, 1992; Urdan and Kaplan, 2020). When students adopt higher levels of achievement goals, their behavioral intentions are oriented toward meeting task standards rather than relying on others to complete the work. Empirical evidence indicates that self-expectation is closely associated with academic motivation and performance. Students with high self-expectation typically demonstrate greater participation and responsibility in group tasks, thereby reducing the occurrence of social loafing (Putri and Dewi, 2021). Furthermore, AGT suggests that different goal orientations elicit distinct affective and behavioral responses. In contexts that emphasize mastery or competence demonstration, learners are more likely to maintain engagement and effort rather than reduce their contribution (Elliot and Sommet, 2023; Zhao, 2025). Therefore, students with high achievement motivation and high self-efficacy are more likely to develop stronger self-expectation in group tasks, which in turn reduces the likelihood of social loafing. This study proposes the following hypothesis:

H3: Self-expectation can inhibit social loafing among university students in group learning.

### 2.6 Peer support

Peer support refers to the emotional, informational, and practical assistance provided by individuals of similar age or experience. This support typically helps others cope with life challenges and difficulties by sharing experiences and offering emotional understanding (Murphy et al., 2023). The primary purpose of peer support is to enhance individuals' confidence, coping abilities, and overall wellbeing through mutual assistance and shared experiences. In the fields of education and health, peer support is widely used to help individuals overcome psychological barriers, improve academic performance, and promote social integration (Murphy et al., 2023). Firstly, peer support significantly improves individuals' mental health by providing emotional support and practical advice. This support can reduce feelings of loneliness and enhance a sense of belonging (Richard et al., 2022). Secondly, research shows that peer support in educational settings can improve students' academic motivation and performance. Through peer-assisted learning, students can better understand course content and enhance their academic outcomes (Sinclair, 2017). Finally, peer support helps enhance individuals' social integration, particularly for those undergoing significant life changes or transitions. By providing a safe and understanding environment, peer support helps individuals better adapt to new circumstances (Payne, 2017).

Peer support can effectively inhibit social loafing behaviors among university students in group learning contexts. From the perspective of AGT, contextual factors within the learning environment influence students' perceptions and adoption of achievement goals (Kaplan and Patrick, 2016; Cheng, 2023). As a contextual motivational source, peer support shapes a goal structure characterized by responsibility and cooperation, thereby influencing behavioral engagement. Empirical evidence supports this relationship. Ferry and Eliana (2017) indicated that peer evaluation is an effective strategy for reducing social loafing. Students in the experimental group demonstrated significantly lower tendencies toward social loafing after receiving peer evaluations, suggesting that peer evaluation encourages more active participation in team tasks. Aggarwal and O'Brien (2008) similarly found that multiple peer evaluations effectively reduce social loafing, increase satisfaction with team members' contributions, and enhance perceptions of fairness in project outcomes. Havard et al. (2023) further reported that assessment-based response strategies reduce social loafing by promoting cooperation and mutual support among students. AGT further suggests that when the learning context emphasizes cooperation and collective growth, students are more likely to adopt adaptive goal orientations that enhance responsibility and task engagement rather than diminish effort (Elliot and Sommet, 2023; Zhao, 2025). Overall, peer support, including peer evaluation and cooperative learning strategies, constitutes an effective mechanism for reducing social loafing behaviors among university students in group learning. This study proposes the following hypothesis:

H4: Peer support can inhibit social loafing among university students in group learning.

### 2.7 Rating fairness

Rating fairness in the context of teachers evaluating students primarily refers to the consistent and impartial methods and standards that teachers use to assess students' performance. These methods should reflect each student's true contributions and abilities without being influenced by external factors such as gender, race, or personal biases (Tierney, 2014). Specifically, fairness includes providing students with equal opportunities to showcase their learning outcomes, ensuring transparency in the evaluation process, maintaining a fair classroom environment, and balancing equality with fairness in treatment. The primary purpose of rating fairness is to ensure that students can learn in a just and unbiased environment, which not only enhances their satisfaction and trust in the learning process but also promotes their learning motivation and engagement (Chai et al., 2024).

Firstly, fair evaluations can stimulate students' learning motivation, encouraging them to participate more actively in learning activities. Research shows that when students perceive evaluations as fair, they are more invested and enthusiastic (Carvalho, 2013). Secondly, in group learning, a fair evaluation mechanism can reduce social loafing behaviors, ensuring that every student fully participates in team tasks and improving overall learning outcomes (Ion et al., 2023). Finally, fair evaluations can enhance students' trust in teachers and the educational system, fostering a supportive and trusting learning environment among students (Wu and Liu, 2020).

Fair evaluations by teachers in group teaching play a crucial role in inhibiting social loafing among university students. When evaluation standards are transparent and applied consistently, students are more likely to perceive that their individual contributions will be fairly recognized. Such perceptions enhance personal accountability and reduce the tendency to decrease effort in collaborative tasks. From the perspective of AGT, evaluation practices constitute an essential part of the classroom goal structure. They shape how students define competence and interpret success (Ames, 1992; Kaplan and Patrick, 2016). When assessment criteria are explicit and perceived as fair, students are more likely to internalize performance standards and regulate their behavior accordingly, rather than relying on others' efforts. In this sense, fair evaluation functions as a situational cue that guides goal-directed behavior and strengthens responsibility. Empirical findings support this argument. Aggarwal and O'Brien (2008) demonstrated that multiple peer evaluations significantly reduce social loafing and increase satisfaction with team members' contributions. Synnott (2016) further indicated that aligning evaluation criteria with authentic team tasks and applying fair standards enhances participation and minimizes slacking behaviors. Similarly, Tien and Dung (2022) found that when students perceive rating fairness, their intentions to share knowledge increase, which further discourages disengagement in group work. AGT also suggests that different achievement structures elicit distinct behavioral and emotional responses (Dever et al., 2024; Urdan and Kaplan, 2020). A fair and well-structured evaluation system clarifies expectations and reinforces effort-based standards, thereby reducing opportunities for social loafing. Taken together, fair evaluation methods in group teaching can effectively inhibit social loafing, foster cooperation, and enhance learning outcomes. This study therefore proposes the following hypothesis:

H5: Rating fairness can inhibit social loafing among university students in group learning.

### 2.8 Learning achievement

Learning achievement refers to the intellectual performance outcomes students achieve in school, college, or university. This includes test scores, completion rates, and other objective measures such as academic grades. Learning achievement reflects students' performance in the educational process and is a crucial indicator of individual intellectual education (Kushwaha and Kumar, 2023). The primary purpose of learning achievement is to promote students' intellectual development and knowledge accumulation, helping them succeed in their future careers. Additionally, learning achievement aims to enhance students' self-efficacy and learning motivation, helping them develop good learning habits and strategies (Yang and Wang, 2022). In higher education, excellent academic performance is often seen as a prerequisite for career success (Arora and Singh, 2017). Finally, learning achievement is a key indicator of educational quality and student success. High academic achievement usually signifies the success of educational institutions in teaching quality and student development (Barros and Simão, 2018).

Social loafing has a significant negative impact on students' academic achievement, particularly in higher education contexts in which collaborative learning is prevalent. When students reduce their individual effort in group tasks, the overall quality of task engagement declines, thereby adversely affecting learning outcomes. Empirical evidence indicates that social loafing diminishes individual contributions and undermines collective performance. Eliana and Novliadi (2020) found that students with higher tendencies toward social loafing tend to exhibit lower academic achievement. Their findings further suggest that strengthening achievement motivation reduces social loafing and enhances academic performance. From the perspective of AGT, learning outcomes depend on the goal orientation adopted by individuals and their subsequent behavioral engagement (Dever et al., 2024). When students decrease their personal effort, their commitment to achievement goals is weakened, resulting in reduced cognitive engagement and strategic regulation, which ultimately impairs academic performance. AGT also posits that achievement goals organize cognitive, emotional, and behavioral processes that directly shape learning outcomes (Bardach et al., 2020; Urdan and Kaplan, 2020). Therefore, when social loafing disrupts sustained effort and goal directed engagement, academic achievement is negatively affected. Taken together, social loafing in group learning is likely to hinder university students' learning achievement. This study therefore proposes the following hypothesis:

H6: Social loafing in group learning among university students negatively affects learning achievement.

Social loafing plays a significant mediating role between self-expectation and academic achievement. Research indicates that self-expectation (such as achievement motivation) can influence students' social loafing behavior, thereby affecting their academic achievement. Specifically, when students have high self-expectation, their tendency for social loafing in group cooperation is lower, which helps improve academic achievement. Eliana and Novliadi (2020) showed that low achievement motivation is associated with a high tendency for social loafing, and a high tendency for social loafing predicts lower academic achievement. This indicates that achievement motivation improves students' academic achievement by reducing social loafing. Students' self-expectation can improve their academic achievement by reducing social loafing. This mediating effect suggests that enhancing students' self-expectation (such as achievement motivation and self-efficacy) is effective in improving academic achievement. This study proposes the following hypothesis:

H7: University students' self-expectation reduces the impact of social loafing on learning achievement.

Social loafing also plays a significant mediating role between peer support and academic achievement. Research indicates that peer support can promote academic achievement, and social loafing mediates this process. Specifically, when students feel supported by their peers, their social loafing behavior decreases, thereby improving academic achievement. Havard et al. (2023) found that promoting cooperation and mutual support among students effectively reduces social loafing behavior, thereby improving educational outcomes. This indicates that peer support can enhance students' academic achievement by reducing social loafing. Furthermore, Wu and Kang (2023) further confirmed the impact of peer support on academic achievement. They found that peer support significantly influences students' academic self-concept, which in turn affects their achievement in learning English as a foreign language. This means that peer support not only directly affects academic achievement but also indirectly promotes academic achievement by enhancing students' self-concept and reducing social loafing. This study proposes the following hypothesis:

H8: University students' peer support reduces the impact of social loafing on learning achievement.

Social loafing also mediates the relationship between rating fairness and academic achievement. Research shows that when students perceive the evaluation system as fair and transparent, their engagement and sense of responsibility in team tasks increase, thereby reducing social loafing and improving academic achievement. Price et al. (2006) found that the fairness of the evaluation system is negatively related to students' social loafing behavior, indicating that when students perceive evaluations as fair, their social loafing behavior decreases. This suggests that a fair evaluation system can enhance students' academic achievement by reducing social loafing. This study proposes the following hypothesis:

H9: University students' perception of rating fairness reduces the impact of social loafing on learning achievement.

**Figure 1:**
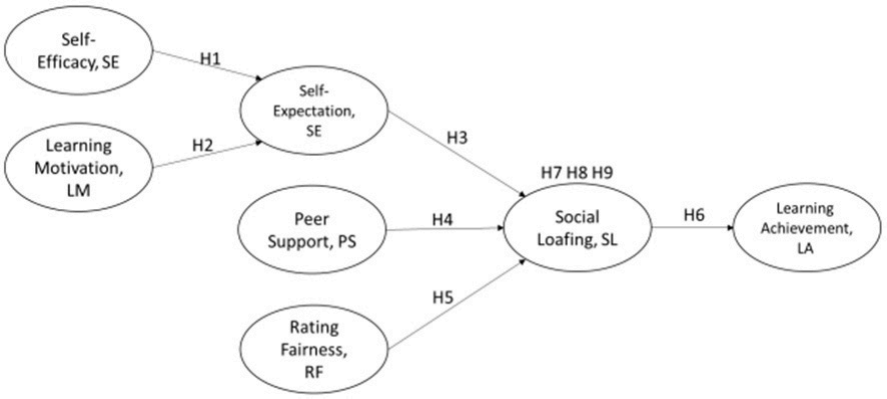
Conceptual model.

Integrating the above hypotheses, the conceptual model of this study is illustrated in Figure 1.

## 3 Research design

### 3.1 Research subjects and data collection

This study aims to explore the mechanisms influencing the learning achievement of university students in group teaching. Therefore, the research subjects are university students. The study adopts a questionnaire survey method, using the platform Wenjuanxing for data collection. A certain number of university students from universities in Shanxi Province were selected to collect research data.

An ethics review committee approved an exemption from a full ethical review for my project on December 15, 2023 (Document No. Quantitative Analysis and Research Association 1121215001). The committee approved the exemption because I conducted a low-risk, non-invasive survey questionnaire. Before administering the survey, I obtained informed consent from all participants. The survey was conducted from January 23, 2024, to January 31, 2024, with a total of 1,387 questionnaires collected. After excluding invalid questionnaires, a total of 600 valid questionnaires were obtained. According to the sample size requirement formula and the recommendations from The Survey System website (https://www.surveysystem.com/sscalc.htm), with a 95% confidence level and a 5% margin of error, the required sample size for the population is 384.

### 3.2 Explanation of research variables

The research variables in this study include the personal background of university students, self-efficacy, learning motivation, self-expectation, peer support, rating fairness, social loafing, and learning achievement. To operationalize the concepts of each research variable, the operational definitions of each variable are described as follows:

#### 3.2.1 Personal background of university students

Since this study primarily explores the mechanisms influencing university students' learning achievement in group-based teaching contexts, the first section of the questionnaire collects participants' personal background information. This section includes four demographic variables: gender, grade level, place of origin, and type of major.

Gender is categorized into two options: male and female. Grade level consists of four options: freshman, sophomore, junior, and senior. Place of origin includes three options: city, town, and rural area. Finally, type of major is classified into three options: liberal arts, science, and arts and physical education.

#### 3.2.2 Design of measurement questionnaire for latent variables

The items are measured using a Likert seven-point scale, ranging from Strongly Disagree (1), Disagree (2), Somewhat Disagree (3), Neutral (4), Somewhat Agree (5), Agree (6), to Strongly Agree (7). Higher scores indicate a higher degree of agreement with the research variables by the respondents. After designing the questionnaire items, experts and scholars are invited to review the items and provide feedback.

Because some of the scales in this study were adapted from different research contexts, the authors invited three scholars with backgrounds in educational research to conduct an expert review after completing the language translation and semantic revisions, in order to ensure that the items could be appropriately transferred to the context of university students' group learning. The three scholars reviewed both the original scale items from the source literature and the revised items used in this study. They evaluated whether the item meanings were clear, whether the items were appropriate for the educational context, whether they reflected the core meanings of the original constructs, and whether they demonstrated content representativeness. Based on the experts' suggestions, the authors made minor wording adjustments to some items to reduce potential comprehension bias caused by differences between the original scale contexts and the research context of this study. Through this procedure, this study confirmed that the adapted scales could still maintain the basic meanings of the original constructs in the group learning context and improve the construct equivalence and content validity of the scales. The design of each construct's questionnaire is described as follows:

#### 3.2.3 Measurement of self-efficacy

For the construct of self-efficacy, this study references the research by Pathak and Joshi (2021), which defines self-efficacy as the confidence individuals show in addressing strategies through meeting discussions during the COVID-19 pandemic, as part of the impact of psychological capital and life satisfaction on organizational resilience. The original questionnaire consists of six items. For example, “During the COVID-19 pandemic, I am confident in analyzing long-term problems to find solutions,” and “During the COVID-19 pandemic, I am confident in communicating with management on behalf of my field in online meetings.” These items were translated and reviewed by experts to ensure consistency with the intended meaning and then adjusted based on the self-efficacy of university students in group teaching. A total of six items were designed, as shown in Table 1.

**Table 1:** Self-efficacy questionnaire items.

Self-efficacy	Reference
1. I am confident in using group learning methods for studying.	Pathak and Joshi, 2021
2. I am confident in communicating with classmates using group learning methods.	
3. I am confident in discussing with classmates using group learning methods.	
4. I am confident in setting learning goals using group learning methods.	
5. I am confident in discussing issues with others using group learning methods.	
6. I am confident in sharing learning information using group learning methods.	

#### 3.2.4 Measurement of learning motivation

For the construct of learning motivation, this study references the research by Luo and Ye (2020). In exploring the learning motivation of university students, items such as “introducing more engaging topics in conversations” and “enhancing personal value and self-fulfillment” were translated and reviewed by experts to ensure consistency with the intended meaning. Based on this, the items were revised, resulting in a total of four questions, as shown in Table 2.

**Table 2:** Learning motivation questionnaire items.

Learning motivation	Reference
1. I find group learning methods more engaging for my studies.	Luo and Ye, 2020
2. I believe group learning is an efficient way to learn.	
3. Through group learning, I have had comfortable and enjoyable learning experiences.	
4. I think group learning is a very good way to learn.	

#### 3.2.5 Measurement of self-expectation

For the construct of self-expectation, this study references the research by Rajeh et al. (2021) on students' expectations of e-learning. Items such as “Using e-learning can improve my academic performance” and “Using e-learning can enhance my learning outcomes” were translated and reviewed by experts to ensure consistency with the intended meaning. Based on this, the items were revised, resulting in a total of three questions, as shown in Table 3.

**Table 3:** Self-expectation questionnaire items.

Self-expectation	Reference
1. Using group learning methods can improve my academic performance.	Rajeh et al., 2021
3. Using group learning methods can enhance my learning outcomes.	
4. I believe that using group learning methods is beneficial for me.	

#### 3.2.6 Measurement of rating fairness

This questionnaire references the items from the study by Roy et al. (2018) on the role of fairness in customer engagement behavior. Items such as “Overall, my service provider is fair to me” and “My service provider keeps its promises” were translated and reviewed by experts to ensure consistency with the intended meaning. Based on this, the items were revised, resulting in a total of four questions, as shown in Table 4.

**Table 4:** Rating fairness questionnaire items.

Rating fairness	Reference
1. Overall, my teacher is fair to me in group learning.	Roy et al., 2018
2. In group learning, my teacher keeps their promises.	
3. In group learning, my teacher treats me with politeness and respect.	
4. In group learning, my teacher treats all students equally.	

#### 3.2.7 Measurement of peer support

For the construct of peer support, this study references the causal model of job satisfaction in different cultural contexts by Chieh-Peng et al. (2003). Items such as “I have colleagues who are willing to provide necessary help at work” and “My colleagues and I always encourage each other” were revised to fit the context of this study, resulting in a total of four questions, as shown in Table 5.

**Table 5:** Peer support questionnaire items.

Peer support	Reference
1. In the study group, there are classmates who are willing to provide me with the necessary help.	Chieh-Peng et al., 2003
2. I feel appreciated by my classmates in the study group.	
3. In the study group, my classmates and I always encourage each other.	
4. My classmates in the study group are always willing to spend time discussing issues with me.	

#### 3.2.8 Measurement of social loafing

This study's questionnaire adopts items from Lam and Campbell (2021) research on the impact of leader relationship management on social loafing. Items such as “Shifts responsibilities they should be performing to other group members” and “Puts in less effort when other group members are present and able to complete the work” were translated and reviewed by experts to ensure consistency with the intended meaning. Based on this, the items were revised and used in this study's questionnaire, as shown in Table 6.

**Table 6:** Social loafing questionnaire items.

Social loafing	Reference
1. In group learning, I sometimes shift responsibilities that I should perform to other group members.	Lam and Campbell, 2021
2. In group learning, I put in less effort when other group members are present and able to complete the work.	
3. In group learning, I sometimes do not do the tasks assigned to me.	
4. In group learning, if other members can complete the work, I spend less time helping.	
5. In group learning, sometimes I put in less effort than other group members.	
6. In group learning, if other group members are present and able to complete the work, I choose easier tasks.	

#### 3.2.9 Measurement of learning achievement

This study's questionnaire adopts items from the research by Pi et al. (2014) on serious leisure, volunteer motivation, and subjective wellbeing in leisure activities. Items such as “Volunteering in this activity has increased my knowledge” and “Volunteering in this activity has enhanced my creativity” were revised for use in this study's questionnaire, as shown in Table 7.

**Table 7:** Learning achievement questionnaire items.

Learning achievement	Reference
1. Group learning has increased my knowledge.	Pi et al., 2014
2. Group learning has enhanced my creativity.	
3. Group learning has strengthened my sense of achievement.	
4. Group learning has enabled me to apply my expertise.	

### 3.3 Data analysis

The data analysis in this study is divided into three parts: descriptive statistical analysis, measurement model validation, and structural equation modeling analysis. First, SPSS was used for descriptive statistical analysis, which included two aspects: calculating the frequency distribution of demographic data to understand the basic characteristics of the sample and calculating the mean and standard deviation of each construct item to understand the central tendency and dispersion of the data. Next, this study validated the measurement model through confirmatory factor analysis, convergent validity, and discriminant validity. The specific steps are as follows: using the statistical software AMOS 24.0 for confirmatory factor analysis. To verify whether the items can adequately measure the variance of the constructs, we conducted a comprehensive assessment of the measurement model. This included an examination of item reliability, construct reliability, and convergent validity. Simultaneously, the average variance extracted (AVE) method was used to test the discriminant validity between constructs, ensuring they are sufficiently distinct from one another. Furthermore, to rule out the potential threat of common method variance (CMV), a single-factor confirmatory factor analysis was performed. The results indicated that a model with all indicators loading on a single common method factor demonstrated significantly poorer fit compared to the hypothesized multi-factor model, suggesting that CMV is unlikely to be a substantial source of bias in this study. Finally, the structural model was further tested, including model fit and hypothesis testing, to confirm the validity and reliability of the research model. Through these steps, this study aims to comprehensively and accurately analyze the data and verify the research hypotheses.

## 4 Research results

### 4.1 Analysis of demographic data

There are 300 female and 300 male participants, each accounting for 50% of the sample. In terms of majors, there are 200 participants from liberal arts, 200 from arts and sports, and 200 from science majors, each accounting for 33.3% (Table 8).

**Table 8:** Analysis of demographic data.

Category	Group	Frequency	Percentage (%)
Gender	Female	300	50
	Male	300	50
Your major belongs to	Liberal Arts	200	33.3
	Arts and Sports	200	33.3
	Science	200	33.3

### 4.2 Convergent validity

Table 9 presents the results of the confirmatory factor analysis for the measurement model. All standardized factor loadings were statistically significant (*p* < 0.001), exceeding the recommended threshold of 0.5, thereby supporting indicator reliability. Composite reliability (CR) values for all constructs were above 0.7, indicating adequate internal consistency. The average variance extracted (AVE) for each construct surpassed 0.5, confirming convergent validity. This signifies that the items collectively explain more than half of the variance in their respective constructs (Fornell and Larcker, 1981). The constructs measured were Self-Efficacy (SE), Learning Motivation (LM), Self-Expectation (SEXP), Peer Support (PS), Resilience Factor (RF), Social Loafing (SL), and Learning Achievement (LA). The results establish a robust and reliable measurement model for testing the proposed hypotheses.

**Table 9:** Results for the measurement model.

Construct	Item	Significance of estimated parameters				Item reliability		Composite reliability	Convergence validity
		Unstd.	S.E.	Unstd./S.E.	p	Std.	SMC	CR	AVE
SE	SE1	1.000				0.888	0.789	0.968	0.834
	SE2	1.031	0.028	36.868	< 0.001	0.929	0.863		
	SE3	1.026	0.026	38.812	< 0.001	0.948	0.899		
	SE4	0.973	0.030	32.666	< 0.001	0.885	0.783		
	SE5	1.005	0.027	36.802	< 0.001	0.930	0.865		
	SE6	0.951	0.028	33.423	< 0.001	0.896	0.803		
LM	LM1	1.000				0.886	0.785	0.954	0.840
	LM2	1.037	0.031	33.289	< 0.001	0.897	0.805		
	LM3	1.049	0.028	36.818	< 0.001	0.935	0.874		
	LM4	1.027	0.027	37.899	< 0.001	0.947	0.897		
SEXP	SEXP1	1.000				0.935	0.874	0.959	0.886
	SEXP2	1.033	0.021	48.509	< 0.001	0.955	0.912		
	SEXP3	1.016	0.023	43.398	< 0.001	0.933	0.870		
PS	PS1	1.000				0.808	0.653	0.908	0.712
	PS2	1.164	0.050	23.134	< 0.001	0.825	0.681		
	PS3	1.170	0.046	25.189	< 0.001	0.876	0.767		
	PS4	1.293	0.052	24.823	< 0.001	0.865	0.748		
RF	RF1	1.000				0.867	0.752	0.957	0.849
	RF2	0.991	0.027	36.325	< 0.001	0.954	0.910		
	RF3	0.936	0.027	35.194	< 0.001	0.943	0.889		
	RF4	0.993	0.030	33.420	< 0.001	0.919	0.845		
SL	SL1	1.000				0.525	0.276	0.830	0.453
	SL2	1.283	0.110	11.700	< 0.001	0.687	0.472		
	SL3	1.007	0.093	10.801	< 0.001	0.611	0.373		
	SL4	1.555	0.140	11.084	< 0.001	0.714	0.510		
	SL5	1.217	0.104	11.696	< 0.001	0.734	0.539		
	SL6	1.530	0.136	11.262	< 0.001	0.739	0.546		
LA	LA1	1.000				0.892	0.796	0.955	0.840
	LA2	1.105	0.029	37.483	< 0.001	0.934	0.872		
	LA3	1.064	0.029	36.314	< 0.001	0.929	0.863		
	LA4	1.083	0.031	34.673	< 0.001	0.911	0.830		

Unstd., Unstandardized factor loadings; Std, Standardized factor loadings; SMC, Square Multiple Correlations; CR, Composite Reliability; AVE, Average Variance Extracted.

SE, Self-Efficacy; LM, Learning Motivation; SEXP, Self-Expectation; SL, Social Loafing; PS, Peer Support; LA, Learning Achievement.

### 4.3 Discriminant validity

Table 10 presents the discriminant validity assessment based on the Fornell-Larcker criterion (Fornell and Larcker, 1981). Discriminant validity is established when the square root of each construct's Average Variance Extracted (AVE, bold diagonal values) exceeds its highest correlation with any other construct (off-diagonal values). The results show this criterion is generally met. For instance, the square root of AVE for Learning Motivation (LM, 0.917) is greater than its highest correlation (0.921 with Self-Expectation, SEXP), though the marginal difference warrants note (Henseler et al., 2015). All other constructs clearly satisfy the criterion. Despite Social Loafing (SL) having a lower AVE (0.453), its square root (0.673) still exceeds all its correlations, supporting its distinctiveness. The overall evidence indicates the constructs in the measurement model are empirically distinct (Hair et al., 2019).

**Table 10:** Discriminant validity for the measurement model.

Construct	AVE	SE	LM	SEXP	PS	RF	SL	LA
SE	0.834	**0.913**						
LM	0.840	0.822	**0.917**					
SEXP	0.886	0.803	0.921	**0.941**				
PS	0.712	0.697	0.740	0.694	**0.844**			
RF	0.849	0.489	0.493	0.466	0.635	**0.921**		
SL	0.453	−0.361	−0.383	−0.373	−0.460	−0.442	**0.673**	
LA	0.840	0.187	0.198	0.193	0.238	0.229	−0.517	**0.917**

The items on the diagonal on bold represent the square roots of the AVE; off-diagonal elements are the correlation estimates.

SE, Self-Efficacy; LM, Learning Motivation; SEXP, Self-Expectation; SL, Social Loafing; PS, Peer Support; LA, Learning Achievement.

### 4.4 Common method variance

This study adopts the CFA comparison method (Mossholder et al., 1998). Model 1: All constructs are merged into one factor structure (that is, all questionnaires are only related to one common factor). Model 2: Theoretically CFA has a completely related structure. If the degree of freedom and chi-square value of the difference between model 1 and model 2 reach significance, which means that the factor structure does not exist, so CMV is not serious. Following the logic of the “single factor procedure” (Organ, 1986), if method variance substantially accounts for the covariation among measures, a confirmatory factor analysis should indicate that a single-factor model fits the data well. The results showed that a model with all indicators loading on a single method factor exhibited poor fit (χ^2^ = 8,461.085, DF= 434, TLI =0.589, CFI =0.616, RMSEA =0.176). In contrast, the original multi-factor CFA model demonstrated good fit (χ^2^ = 1,358.249, DF = 413, TLI =0.949, CFI =0.955, RMSEA =0.062). The difference between the models was statistically significant (Δχ^2^ = 7,102.836, ΔDF = 21, *p* < 0.001). These results indicate that common method variance is unlikely to be a serious threat to the relationships among the measured variables in this study (Table 11).

**Table 11:** CMV test of model comparison.

Model	Model fit					Model different comparison		
	χ^2^	DF	TLI	CFI	RMSEA	Δχ^2^	ΔDF	*p*
CMV model	8,461.085	434	0.589	0.616	0.176	7,102.836	21	< 0.001
Original CFA model	1,358.249	413	0.949	0.955	0.062			

### 4.5 Goodness of fit

Table 12 presents the overall fit indices for the research model. The results indicate a mixed pattern of model fit. While several key incremental and absolute fit indices met or exceeded their respective benchmarks—including the Tucker-Lewis Index (TLI = 0.923), Comparative Fit Index (CFI = 0.930), Goodness-of-Fit Index (GFI = 0.912), and Adjusted Goodness-of-Fit Index (AGFI = 0.903), all surpassing the recommended threshold of 0.90 (Hu and Bentler, 1999). The Normed Chi-square (χ^2^/DF = 4.468) exceeded the acceptable range of 1 to 5 (Schumacker and Lomax, 2004). However, the Root Mean Square Error of Approximation (RMSEA = 0.076) fell within the acceptable range (< 0.08; Browne and Cudeck, 1993). Given that CFI and TLI are less sensitive to sample size than χ^2^, and considering the acceptable RMSEA, the model demonstrates reasonable, though not excellent, fit to the data. Researchers should interpret the structural relationships with caution, acknowledging the elevated SRMR and χ^2^/DF values.

**Table 12:** Model fit.

Model fit	Criteria	Model fit of research model
MLχ^2^	The small the better	1,885.464
DF	The large the better	422.000
Normed Chi-sqr (χ^2^/DF)	1 < χ^2^/DF < 5	4.468
RMSEA	< 0.08	0.076
TLI (NNFI)	>0.9	0.923
CFI	>0.9	0.930
GFI	>0.9	0.912
AGFI	>0.9	0.903

### 4.6 Path analysis

**Figure 2:**
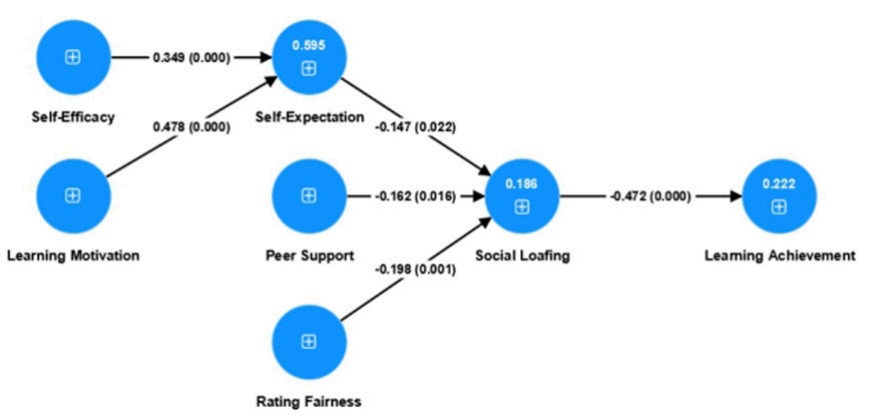
PLS-Sem statistical model diagram.

Table 13 and Figure 2 presents the structural model results testing the hypothesized relationships. Self-Expectation (SEXP) was significantly predicted by both Self-Efficacy (SE; β = 0.142, *p* < 0.001) and Learning Motivation (LM; β = 0.804, *p* < 0.001), with LM being the dominant predictor. Together, these variables explained a substantial proportion of variance in SEXP (*R*^2^ =0.854). Social Loafing (SL) was significantly negatively predicted by Peer Support (PS; β = −0.240, *p* =0.002) and Reflection (RF; β = −0.248, *p* < 0.001), but not significantly by Self-Expectation (SEXP; β = −0.090, *p* =0.156). This model explained 25.4% of the variance in SL. Finally, Learning Achievement (LA) was significantly negatively predicted by Social Loafing (SL; β = −0.517, *p* < 0.001), with SL accounting for 26.7% of the variance in LA. These findings highlight the strong role of motivation in shaping expectations, the protective role of peer support and reflection against social loafing, and the detrimental impact of social loafing on academic performance.

**Table 13:** Regression coefficient.

DV	IV	Unstd	S.E.	Unstd./S.E.	*p*-value	Std.	*R* ^2^
SEXP	SE	0.149	0.039	3.833	< 0.001	0.142	0.854
	LM	0.777	0.040	19.342	< 0.001	0.804	
SL	SEXP	−0.046	0.033	−1.417	0.156	−0.090	0.254
	PS	−0.180	0.058	−3.119	0.002	−0.240	
	RF	−0.157	0.037	−4.250	< 0.001	−0.248	
LA	SL	−0.818	0.092	−8.905	< 0.001	−0.517	0.267

SEXP, Self-Expectation; SL, Social Loafing; PS, Peer Support; LA, Learning Achievement.

### 4.7 Mediation effects

Table 14 presents the results of the mediation analysis examining indirect effects via Social Loafing (SL). Using bias-corrected bootstrapping with 1,000 samples, the indirect effect from Self-Expectation to Learning Achievement through SL was not statistically significant (point estimate = 0.038, 95% CI [−0.020, 0.102], *p* =0.221). In contrast, the indirect effects from both Peer Support (point estimate = 0.147, 95% CI [0.041, 0.263], *p* =0.010) and Reflection (point estimate = 0.128, 95% CI [0.070, 0.194], *p* < 0.001) to Learning Achievement through SL were significant. Specifically, higher levels of Peer Support and Reflection were associated with lower levels of Social Loafing, which in turn predicted higher Learning Achievement. This result indicates that SL serves as a significant mediator for the effects of PS and RF on LA, while the mediating role of SL in the relationship between SEXP and LA is not supported. The findings underscore the importance of fostering supportive peer interactions and reflective practices to mitigate social loafing and subsequently enhance academic outcomes.

**Table 14:** The analysis of indirect effects.

Effect	Point estimate	product of coefficients			Bootstrap 1000 times Bias-corrected 95%	
		S.E.	*Z*-value	*p*-value	Lower bound	Upper bound
SEXP → F0E0SL → F0E0LA	0.038	0.031	1.224	0.221	−0.020	0.102
PS → F0E0SL → F0E0LA	0.147	0.057	2.576	0.010	0.041	0.263
RF → F0E0SL → F0E0LA	0.128	0.033	3.887	< 0.001	0.070	0.194

SEXP, Self-Expectation; SL, Social Loafing; PS, Peer Support; LA, Learning Achievement.

## 5 Discussion

The relationships among achievement goals, social loafing, and learning achievement in group teaching contexts are complex and intertwined, highlighting the need for theoretical clarification within higher education settings. To examine these relationships, this study applied AGT as the foundational framework and employed structural equation modeling to test the proposed paths and mediating mechanisms. The findings provide several important interpretations grounded in the theoretical logic of achievement goal orientation and classroom goal structure.

According to AGT, achievement goals shape how individuals interpret competence, responsibility, and standards of success, thereby influencing their behavioral engagement in group contexts (Urdan and Kaplan, 2020). In this study, self-efficacy, learning motivation, and self-expectation were conceptualized as mastery-oriented internal factors, whereas peer support and rating fairness represented contextual goal structures. The results indicate that mastery-oriented factors did not significantly reduce social loafing. This outcome can be explained by the core emphasis of mastery goals on personal growth and understanding (Noordzij et al., 2021). Such goals are primarily self-referenced and internally regulated, focusing on competence development rather than social accountability. This finding can also be understood from the perspective of cultural and institutional factors. Because the sample of this study consisted of university students from Shanxi Province, their group learning experiences may have been simultaneously influenced by grade orientation, awareness of ranking, and norms of collective relationships. Xu (2024) indicated that Chinese university students engage in strategic adaptation among high-stakes academic performance, teacher requirements, policy orientations, and existing learning cultures. Therefore, students' engagement in group tasks depends not only on whether they possess mastery goals but also on whether individual contributions can be identified, whether responsibilities are clearly defined, and whether the grading system is perceived as fair. This also echoes Lin et al. (2025) view that cultural values may influence achievement goals and learning achievement.

In group tasks where individual contributions are less visible, internal growth orientation alone may not sufficiently counteract responsibility diffusion. In contrast, contextual factors such as peer support and perceived rating fairness significantly reduced social loafing. AGT suggests that classroom goal structures influence how students define competence and interpret evaluation criteria (Boden et al., 2020; Cheng, 2023). When evaluation standards are transparent and perceived as fair, students are more likely to connect effort with outcomes and strengthen personal accountability. Empirical findings by Aggarwal and O'Brien (2008) and Ion et al. (2023) demonstrate that fair and multiple evaluation systems enhance contribution visibility and reduce loafing tendencies. Similarly, cooperative and supportive peer environments foster responsibility norms and mutual expectations (Havard et al., 2023). These performance-related contextual cues exert stronger influence on observable group behavior than internal mastery orientation alone.

The negative effect of social loafing on learning achievement is also consistent with AGT. The theory posits that achievement goals organize cognitive, emotional, and behavioral processes that directly shape learning outcomes (Bardach et al., 2020). Learning achievement depends not only on ability but also on sustained goal commitment, self-regulation, and effort investment (Dever et al., 2024; Gao and Brown, 2023). Social loafing represents a withdrawal of effort and a weakening of goal-directed engagement, which leads to reduced cognitive processing depth and diminished strategic learning. Elliot and Sommet (2023) note that goal structures elicit distinct emotional and behavioral responses. When students disengage from active participation and attribute success to others' efforts, the personal meaning of achievement goals becomes diluted. Consequently, lower levels of social loafing indicate stronger goal commitment and consistent task engagement, which contribute to higher learning achievement.

The mediating role of social loafing indicates that contextual motivational factors influence learning achievement indirectly through behavioral engagement within group processes. For university students in Shanxi Province participating in group-based instruction, learning outcomes are shaped not solely by internal psychological traits but by how classroom structures regulate responsibility and participation. AGT emphasizes that situational cues guide goal adoption and behavioral regulation (Boden et al., 2020; Cheng, 2023). When students perceive peer support and rating fairness, they are more likely to maintain accountability and sustained involvement, thereby reducing social loafing and improving academic performance. Ion et al. (2023) also indicated that self-assessment and peer assessment can enhance students' perceptions of grading fairness in group assignments, because such institutional arrangements prevent assessment responsibility from being held entirely by teachers and help distinguish individual contributions within group tasks. The mediation effect therefore reflects a behavioral transformation mechanism through which contextual goal structures translate motivational conditions into concrete learning outcomes within collaborative higher education environments.

### 5.1 Theoretical contributions

This study extends both AGT and the literature on social loafing at the theoretical level. First, within the development of AGT, prior research has primarily focused on the direct effects of mastery goals and performance goals on individual learning motivation, self-regulation, and academic achievement, with limited attention to their behavioral transformation mechanisms within team interaction processes. By incorporating social loafing into the theoretical framework, this study demonstrates that achievement goals do not directly determine learning outcomes but instead exert their influence through the level of effort investment within group contexts. This approach expands the explanatory scope of AGT in collaborative learning settings. In addition, this study differentiates between intrinsic mastery orientation and contextual goal structures, revealing that contextual cues, such as peer support and evaluation fairness, exert a more direct influence on behavioral engagement. This finding supplements prior research that has predominantly emphasized individual psychological orientations.

With respect to the study of social loafing, existing literature has largely examined the phenomenon from organizational behavior or social psychological perspectives, focusing on structural factors such as diffusion of responsibility and group size. This study situates social loafing within the framework of AGT and conceptualizes it as a manifestation of weakened goal commitment and reduced behavioral engagement. This perspective shifts the interpretation of social loafing from a purely efficiency related issue to a motivational and goal process issue, thereby linking it to educational psychology theory. Through the construction of an integrated model, this study strengthens the theoretical applicability of AGT to team-based learning contexts and provides a more motivation oriented explanatory foundation for understanding collaborative learning effectiveness in higher education.

### 5.2 Practical contributions

The findings of this study have important implications for university instructors who teach through collaborative groups. Peer evaluation and social loafing appear to be mitigated as a result of peer support, with the end result being improved levels of learning achievement. Therefore, instructors who design collaborative learning courses should consider the importance of having transparent evaluation methods and allow for identification of individual contributions to the group. For the institution itself, collaboration should not just be viewed as an opportunity for instructional innovation, but rather should also result in a supportive environment for learners and in the development of fair assessment standards, in an effort to minimize diffusion of responsibility through institutional design. For students, these results stress the importance of remaining actively engaged in group learning and of being personally accountable for their actions as a member of a group. When students acknowledge that the way they behave impacts the way the group as a whole performs, they are likely to assume responsibility and enhance overall learning. In summary, the findings of this study indicate possible directions for the planning and implementation of collaborative learning in postsecondary education.

### 5.3 Limitations and future research

Although this study revealed the relationships among self-efficacy, learning motivation, self-expectation, peer support, evaluation fairness, social loafing, and academic achievement through empirical analysis, several limitations remain. First, the sample of this study was primarily drawn from a specific educational context and student population, and the findings may not be universally generalizable. Students from different cultural backgrounds, academic disciplines, and educational levels may exhibit different behaviors and attitudes; therefore, the generalizability of the findings is limited. In addition, variables such as social loafing and learning motivation in this study are complex and multidimensional constructs. Future research may further refine the dimensions of these variables to examine the specific effects of each dimension on academic achievement. Beyond the variables examined in this study, future research may also explore other factors that may influence social loafing and academic achievement, such as teaching style, learning environment, and students' personality traits. This would contribute to the development of a more comprehensive theoretical framework and provide guidance for educational practice.

## 6 Conclusion

This study adopts AGT as the core theoretical framework to examine the relationships among motivational factors, social loafing, and learning achievement among university students in the context of group teaching. AGT posits that individuals' understanding of competence and their definition of success shape their behavioral engagement and learning outcomes. This study extends this theory to the context of collaborative learning, indicating that in group settings, learning outcomes are not determined solely by individual intrinsic motivation but are realized through behavioral performance within the process of group interaction. Self-efficacy and learning motivation significantly enhance self-expectation, indicating that intrinsic motivation strengthens students' beliefs regarding learning outcomes. However, self-expectation does not directly suppress social loafing, reflecting that in group tasks, mastery orientation alone is insufficient to counteract the diffusion of responsibility. In contrast, contextual factors such as peer support and evaluation fairness effectively reduce social loafing, demonstrating that classroom goal structures and evaluation systems exert a critical influence on students' behavioral engagement. Furthermore, social loafing has a significant negative effect on learning achievement, confirming that when students reduce their effort and participation, their cognitive engagement and learning outcomes are weakened. Overall, the findings demonstrate that in higher education group teaching, the formation and enactment of achievement goals are strongly regulated by contextual cues, and social loafing serves as a crucial behavioral mechanism linking motivation and learning achievement.

## Data Availability

The raw data supporting the conclusions of this article will be made available by the authors, without undue reservation.
